# Mesenchymal stem cell therapy for paraquat poisoning: A systematic review and meta-analysis of preclinical studies

**DOI:** 10.1371/journal.pone.0194748

**Published:** 2018-03-22

**Authors:** Fang He, Aiting Zhou, Shou Feng, Yuxiang Li, Tao Liu

**Affiliations:** 1 Key Laboratory of Cell Engineering of Guizhou Province, The Affiliated Hospital of Zunyi Medical College, Zunyi, Guizhou, PR China; 2 Department of Spine Surgery, The Affiliated Hospital of Zunyi Medical College, Zunyi, Guizhou, PR China; 3 Beijing Hospital of Traditional Chinese Medicine, Capital Medical University, Beijing Institute of Traditional Chinese Medicine, Beijing, PR China; Università degli Studi della Campania, ITALY

## Abstract

**Background:**

Paraquat (PQ) poisoning can cause multiple organ failure, in which the lung is the primary target organ. There is currently no treatment for PQ poisoning. Mesenchymal stem cells (MSCs), which differentiate into multiple cell types, have generated much enthusiasm regarding their use for the treatment of several diseases. The aim of this study was to systematically review and analyze published preclinical studies describing MSC administration for the treatment of PQ poisoning in animal models to provide a basis for cell therapy.

**Methods:**

The electronic databases PubMed and CBMdisc were searched in this systematic review and meta-analysis. The MSC treatment characteristics of animal models of PQ poisoning were summarized. After quality assessment was performed, the effects of MSC transplantation were evaluated based on the survival rate, lung wet/dry weight, fibrosis scores, oxidative stress response, and inflammatory response. Publication bias was assessed.

**Results:**

Eleven controlled preclinical studies involving MSC transplantation in animal models of PQ poisoning were included in this review. MSC therapy improved the survival rate and reduced the lung wet/dry weight and histopathological fibrosis changes in most studies. MSCs decreased serum or plasma malondialdehyde levels in the acute phase after 7 and 14 d and increased serum or plasma superoxide dismutase and glutathione levels at the same time points. IL-1β, TNF-α and TGF-β1 levels in blood or lung tissues were decreased to different degrees by MSCs. Lung hydroxyproline was decreased by MSCs after 14 d. No obvious evidence of publication bias was found.

**Conclusion:**

MSCs showed anti-fibrosis therapeutic effects in animal models of lung injury caused by PQ poisoning, which may be related to reduced oxidative stress and inflammatory cytokine levels. Our review indicates a potential therapeutic role for MSC therapy to treat PQ poisoning and serves to augment the rationale for clinical studies.

## Introduction

Paraquat (1,1-dichloro-4,4-bipyridine, PQ) is a quaternary nitrogen herbicide used worldwide that exerts strong toxic effects on humans and animals [1]. Due to its low price and easy access, PQ has become a familiar cause of death by pesticide poisoning in developing countries. The lethal dose of PQ is 5–15 ml of water solution containing 20% PQ (20–40 mg/kg) in adults. The fatality rate of PQ poisoning is as high as 50–70% [2]. PQ absorbed through the digestive tract, skin and respiratory tract can cause multiple organ damage, in which the lung is the primary target organ [3–5]. It is thought that the structural similarity of PQ to lung diamines and polyamines such as putrescine, spermine, and spermidine induces the lung to accumulate PQ competitively [6]. The pathologic manifestations of PQ poisoning are pulmonary acute inflammatory infiltration and the rapid development of fibrosis. In clinical practice, there is no specific treatment for PQ poisoning other than minimizing its absorption and attempting to prevent organ injury.

Mesenchymal stem cells (MSCs) are a class of multipotent adult stem cells characterized by both self-proliferation and highly plasticity, unlike immortalized cell lines, which are not stem cells [7]. MSCs differentiate into mesodermal-derived tissues, such as adipocytes, osteoblasts, and chondrocytes, in vitro and in vivo [8] and secrete a variety of cytokines. MSCs are derived from many sources, including bone marrow, umbilical cord blood, dental pulp, adipose tissue and adult organs [9]. These cells exhibit fibroblastic morphology, are generally isolated via adherence to a plastic surface and share a common immuno-phenotype consisting of CD105, CD73 and CD90 expression; they are also negative for CD45, CD34, CD14, CD19 and HLA-DR expression [10]. MSCs showing low immunogenicity are used for xenogenous transplantation to achieve immunomodulation and improve tissue repair. They have also been evaluated for their regenerative potential to treat conditions such as myocardial infarction [11], certain neurodegenerative disorders [12] and chronic lung diseases [13]. Studies have shown that bone marrow and umbilical cord blood-derived MSCs selectively home to sites of lung injury through the stromal derived factor-1/chemokine receptor CXCR4 signaling pathway, differentiate into cells expressing lung epithelial markers and exert paracrine functions [14,15]. MSCs exhibit the potential to improve lung function in bleomycin and lipopolysaccharide pulmonary disease models and are being exploited in the clinic for their therapeutic potential in the treatment of pulmonary fibrosis and pulmonary hypertension [13,16].

The aim of this systematic review and meta-analysis was to investigate the effects of MSCs derived from different sources for the treatment of lung injury induced by PQ. The results obtained from the present review may serve as a reference for the assessment of MSC therapy translation in clinical trials.

## Materials and methods

This systematic review and meta-analysis followed the Preferred Reporting Items for Systematic Reviews and Meta-analyses (PRISMA) criteria [17], which are briefly described here (S1 Table). Additionally, we deposited our laboratory protocols at protocols.io; the identifier is dx.doi.org/10.17504/protocols.io.mwqc7dw.

### Literature search

The PubMed and CBMdisc databases were searched with a date range starting on December 12, 1961; the searches were last updated on September 26, 2017. Medical subject headings (MeSH) combined with individual words were used to select the search terms. Terms used in the search included “Mesenchymal Stem Cell”, “Paraquat” and “Pulmonary” (refer to S4 Table). We also searched the reference lists of the retrieved articles to identify any additional studies that were missed.

### Study selection

All experiments evaluated animals with PQ poisoning treated with normal MSCs alone and a PQ damage group as a control. Reviews and repeated studies were excluded.

### Data extraction and screening

All studies were read, and all data were extracted independently by two reviewers. Disagreements were resolved by a third reviewer. The following study characteristics were extracted from each article: the first author’s name; the year of publication; the sample size of the PQ control group and MSC experimental group; the source, dose, time and delivery route of MSCs; the dose and delivery route of PQ; the species of the recipient animal; and time at outcome assessment. According to the literature [7,8,10], MSC characterization criteria and general methodology in the included studies were also collected and compared. Parameters including changes in survival rate, the lung wet/dry weight ratio, fibrosis scores, and malondialdehyde (MDA), superoxide dismutase (SOD), glutathione (GSH), glutathione peroxidase (GSH-PX), interleukin-1 beta (IL-1β), tumor necrosis factor alpha (TNF-α), and transforming growth factor beta (TGF-β1) levels were reviewed via systematic description. In addition, MDA, SOD, and GSH levels in serum or plasma (insufficient statistical data were excluded) were chosen as functional indices to evaluate the therapeutic effects of MSCs through meta-analysis.

### Risk of bias

Two reviewers (FH and ATZ) assessed the risk of bias in each experiment using SYRCLE's Risk of Bias tool [18], which is based on the Cochrane Risk of Bias tool for animal intervention studies. In cases with discrepancies, a third investigator (SF) was requested to discuss the different opinions. Ten assessment items related to selection, performance, detection, attrition, reporting and other biases in the SYRCLE tool were assessed and scored as low, high, or unclear risk of bias. The questions in the tool had responses of “yes”, “no” or “do not explicitly state”, corresponding to “low risk of bias”, “high risk of bias” or “unclear”, respectively.

### Statistical analysis

Meta-analysis was conducted using Review Manager Version 5.3 (Cochrane collaboration) to generate forest plots and funnel plots. The following data items were used for data entry: (1) mean, standard deviation, and number of animals in the PQ or MSC administration group; and (2) the P value between the two groups. The data were pooled to estimate the mean difference and corresponding 95% confidence intervals. Heterogeneity between studies was assessed with a Chi-squared-based Q test and I^2^. P<0.1 or I^2>^50% denotes significant heterogeneity. Because of the large heterogeneity of the total data set, subgroup analysis of comparisons was performed. Outcomes measured 3, 7, 14 and 21 d after transplantation were extracted separately and used for stratified comparisons. The significance of pooled estimates was assessed with a Z test, in which P<0.05 was considered significant. The presence of publication bias and small-study effects were evaluated and explored using funnel plots. Data unsuitable for quantitative analysis were evaluated by statistical qualitative analysis.

## Results

### Included studies and their characteristics

**Fig 1:**
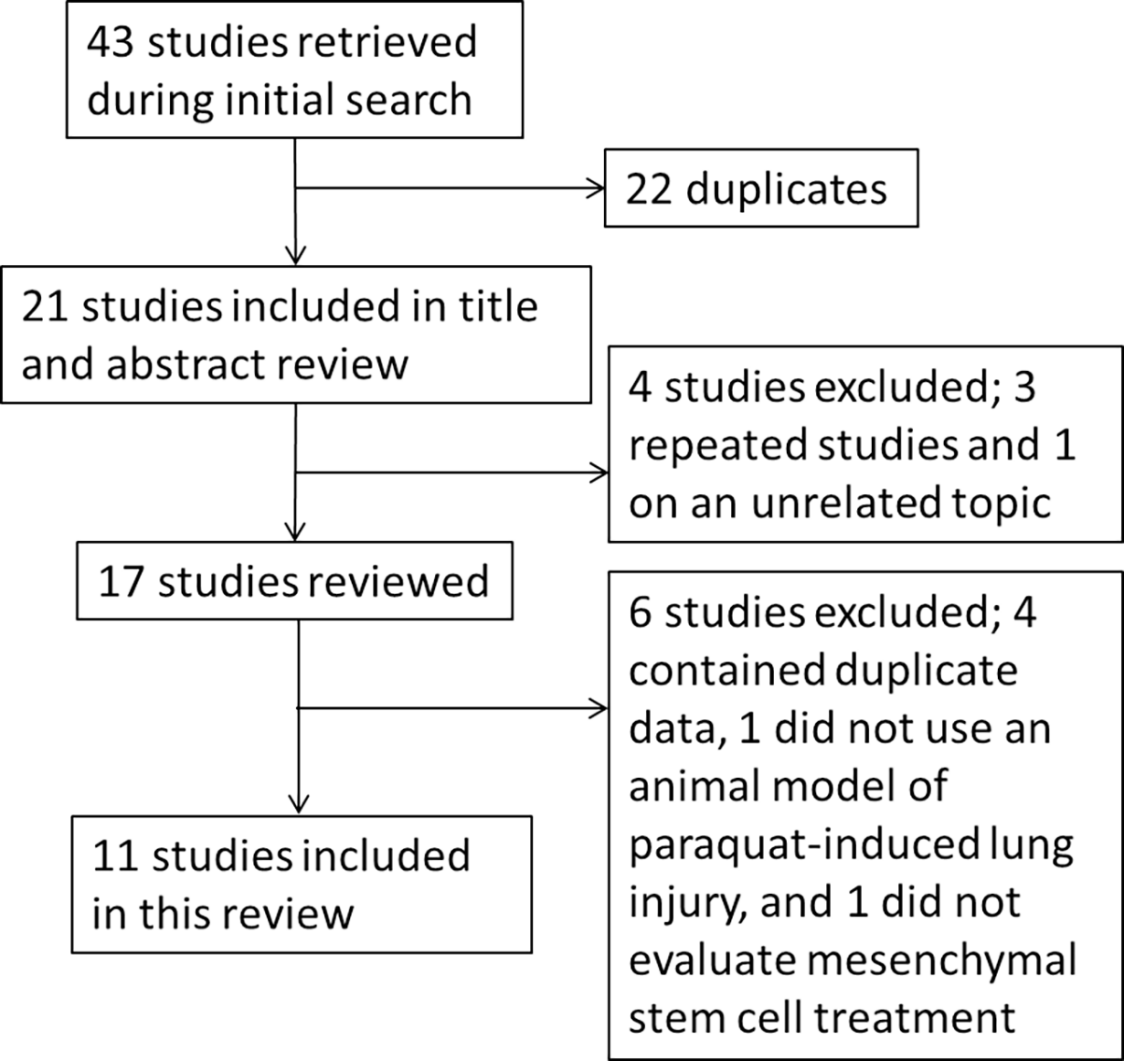
Included and excluded studies.

We identified 43 articles in our search, and only 11 articles met the selection criteria for inclusion in this review (Fig 1). The first authors of the included studies are all from China, and the articles were published from 2011 to 2017. All articles included a PQ poisoning group and an MSC treatment group. Sprague-Dawley rats, Balb/c mice and Wistar rats were used in the studies. Different doses of PQ were used to induce lung injury by intraperitoneal injection or intragastric administration. MSCs were obtained from the bone marrow, umbilical cord and adipose tissue. The dose given varied from 0.1×10^6^ to 10×10^6^ cells, although one study did not indicate the specific dose used. The MSCs were mostly administered intravenously, but two studies used retrobulbar injection. All results in these studies were observed within 30 d (Table 1).

**Table 1:** Characteristics of the included studies.

First author	Year	Recipient animals (MSC/PQ)	MSC source	MSC dose (1×10^6^)/time (after PQ)/delivery method	PQ dose (delivery method)	Time at outcome measurement
Xiong Jianfei	2014	SD rats (24/24)	SD rat bone marrow	1/6 h/i.v.	20% PQ for 15 mg/kg (i.p.)	3, 7, 14 d after MSC transplantation
Gao Jing	2011	Wistar rats (9/9)	Wistar rat bone marrow	0.1/?/i.v.	20% PQ for 18 mg/kg (i.p.)	72 h after MSC transplantation
Chen Min	2016	SD rats (18/18)	Bone marrow	1/6 h/i.v.	20 mg/ml PQ for 120 mg/kg (i.g.)	7, 14, 28 d after PQ administration
Huang Yang	2012	SD rats (15/15)	SD rat bone marrow	10/6 h/i.v.	20% PQ for 5 mg/kg (i.p.)	1, 3, 7 d after MSC transplantation
Huang Yang	2013	SD rats (24/24)	Rat bone marrow	10/6 h /i.v.	200 g/L PQ for 20 mg/kg (i.p.)	1, 3, 7, 14 d after PQ administration
Zhang Yanmin	2011	Wistar rats (20/20)	Wistar rat bone marrow	1/4 h/i.v.	20% PQ for 18 mg/kg (i.p.)	28 d after PQ administration
Wu You	2016	Balb/c mice (24/24)	Balb/c mouse bone marrow	1/6 h/retrobulbar injection	20% PQ for 25 mg/kg (i.p.)	3, 7, 14, 21 d after PQ administration
Lu Shuanghong	2014	Balb/c mice (29/30)	Human umbilical cord	?/24 h/i.v.	40 mg/kg (i.p.)	7, 21 d after MSC transplantation
Liu Hong	2016	Balb/c mice (20/20)	Bone marrow	1/4 h/retrobulbar injection	20% PQ for 25 mg/kg (i.p.)	3, 7, 14, 21 d after MSC transplantation
Wu Lin	2017	C57BL/6 mice (15/15)	Mouse adipose tissue	1/6 h/i.v.	20% PQ for 0.02 L/kg (i.p.)	12, 24, 48 h after MSC transplantation
Hsin-Lin Tsai	2013	SD rats (7/7)	Human bone marrow	5/6 h/i.v.	24 mg/kg (i.p.)	30 d after PQ administration

PQ: paraquat; MSC: mesenchymal stem cell; i.g: intragastric administration; i.v: intravenous administration; i.p: intraperitoneal injection

### Risk of bias

Only one of the studies was judged as having a high risk of bias for all data entries assessed (S2 Table). The MSC and PQ groups were similar at baseline in all studies. Allocation of subjects to MSC and PQ groups in all studies was random, and two studies expressly described the method used for random sequence generation. None of the studies described the method used to conceal allocation, whether animals were randomly housed, whether caregivers and examiners were blinded and the method for outcome assessment by random selection. Blinding of the outcome assessor was described in nine studies and was not described in one study. Only one study was judged as having a high risk for outcome blinding. All studies were assessed as having a low risk of attrition and reporting bias. None of the studies had other problems that might lead to a high risk of bias.

### MSC characteristics

The criteria and general methodology for MSCs used in the studies are summarized in S3 Table. Eight of the studies reported that the MSCs were separated in the lab. The plastic adherence of MSCs was reported in all studies except for one. Positive and negative markers specific to MSCs were detected in seven studies and were not reported in four studies. The mesodermal-derived tissue differentiation capability of MSCs was tested in four studies. Dulbecco’s modified Eagle’s medium ± fetal bovine serum were conventionally used in cell expansion media, with the exception of one study that did not report the medium used. Two studies reported the use of antibiotic solution in the culture medium. The MSC passage number was reported in eight studies; passage numbers ranged between 3 and 13.

### The overall effects of MSCs on PQ-induced lung injury in animals

Survival rates were mentioned in three studies [19–21]. MSCs improved the mean survival time or final survival rate of PQ-poisoned rats. The lung wet/dry weight ratio was measured in 3 studies [22–24]. MSCs reduced the lung wet/dry weight ratio at 72 h and 7 d after transplantation, but no significant differences were observed at 1, 3 and 14 d. Pulmonary fibrosis was assessed in 3 studies [20,21,25]. The Szapiel method and Ashcroft method were used in the studies by Chen et al. [12] and Lü et al. [20], while the study by Tsai et al. [21] did not describe the details of this assessment (Table 2).

**Table 2:** Summary of the major experimental results.

First author	Year	Survival rate	Lung wet/dry weight	Lung fibrosis score	MDA	SOD	GSH	GSH-PX	IL-1β	TNF-α	TGF-β1	HYP
Xiong Jianfei	2014	↑			Plasma NS (3, 14 d), ↓ (7 d)			Plasma ↓ (3 d), ↑ (7 d), NS (14 d)	Plasma NS (3, 14 d), ↓ (7 d)	Plasma NS (3, 14 d), ↓ (7 d)		
Gao Jing	2011		↓ (72 h)		↓ ^a^(72 h)	↑ ^a^(72 h)		↑ ^a^(72 h)				
Chen Min	2016			↓ ^c^(7, 14, 28 d)							↓ ^b^(7, 14, 28 d)	Lung NS (7 d), ↓ (14, 28 d)
Huang Yang	2012		NS (1, 3 d), ↓ (7 d)		Plasma NS (1 d), ↓ (3, 7 d)					Plasma NS (1 d), ↓ (3, 7 d)		
Huang Yang	2013		NS (1, 3, 14 d), ↓ (7 d)		Plasma NS (1, 14 d), ↓ (3, 7 d)	Plasma NS (1, 14 d), ↓ (3, 7 d)			Plasma ↓ (1, 3, 7 d), NS (14 d)	Plasma ↓ (1, 3, 7 d), NS (14 d)		
Zhang Yanmin	2011										Serum ↓ (28 d)	Lung ↓ (28 d)
Wu You	2016				Serum ↓ (3, 7, 14 d), NS (21 d)	Serum ↑ (3, 7, 14, 21 d)	Serum ↑ (3, 7, 14, 21 d)		Serum ↓ (3, 7, 14, 21 d)	Serum ↓ (3, 7, 14, 21 d)		
Lu Shuanghong	2014	↑		↓ ^d^(21 d)								
Liu Hong	2016				Serum ↓ (3, 7, 14, 21 d)	Lung protein ↑ (3, 21 d); serum ↑ (3, 7, 14 d), NS (21 d)	Serum ↑ (3, 7, 21 d), NS (14 d)			Serum NS (3 d), ↓ (7, 14, 21 d)	Serum ↓ (3, 7, 14 d), NS (21 d)	
Wu Lin	2017								Plasma NS (12, 24 h), ↓ (48 h)	Plasma NS (12, 24 h), ↓ (48 h)		
Hsin-Lin Tsai	2013	↑		↓ ^e^(30 d)								

↑: increased compared with the PQ group (<0.05)

↓: decreased compared with the PQ group (<0.05); NS: not significant; MDA: malondialdehyde; SOD: superoxide dismutase; GSH: glutathione; GSH-PX: glutathione peroxidase; HYP: hydroxyproline

a Lung tissue homogenate

b Lung immunohistochemical semiquantitative analysis

c Szapiel method

d Ashcroft method

e Did not indicate the method

### The effects of MSCs on the oxidative stress response

**Fig 2:**
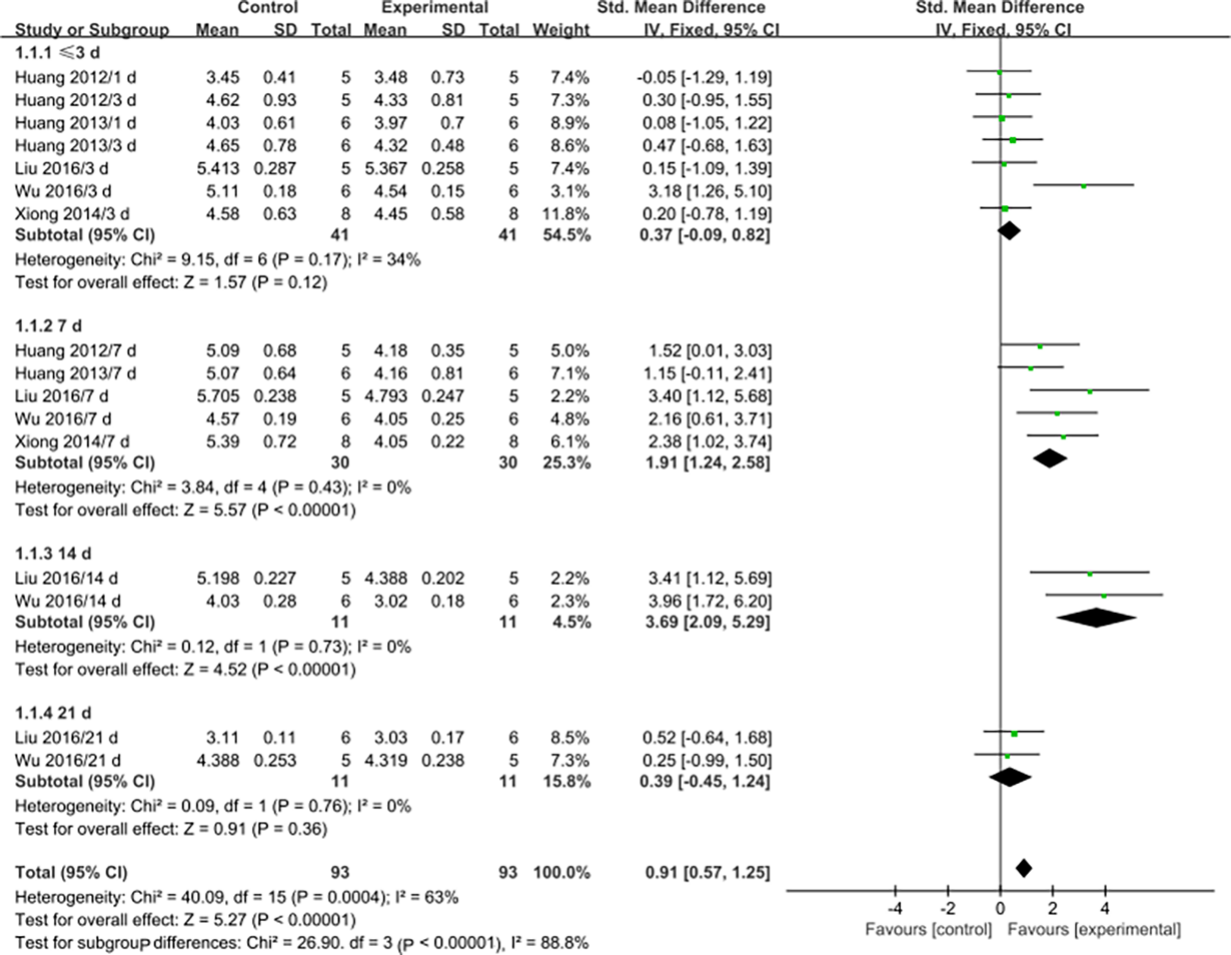
Forest plots showing the effects of MSCs on MDA levels. Control: PQ group; Experimental: MSC treatment group; CI: confidence interval; IV: independent variable; SD: standard deviation.

Serum or plasma MDA levels were assessed at 3 d after MSC transplantation or earlier in seven studies [19,23,24,26,27], at 7 d after transplantation in five studies [19,23,24,26,27], at 14 d after transplantation in two studies [26,27], and at 21 d after transplantation in two studies [26,27] (Table 2). Generally, MSC therapy was associated with significantly decreased MDA levels at 7 d (SMD: 1.91, 95% CI: 1.24 to 2.58, P<0.00001) and 14 d after transplantation (SMD: 3.69, 95% CI: 2.09 to 5.29, P<0.00001), but not at 3 d or earlier (SMD: 0.37, 95% CI: -0.09 to 0.82, P = 0.12) or 21 d after transplantation (SMD: 0.39, 95% CI: - 0.45 to 1.24, P = 0.36). No significant heterogeneity was observed in the groups (Fig 2).

**Fig 3:**
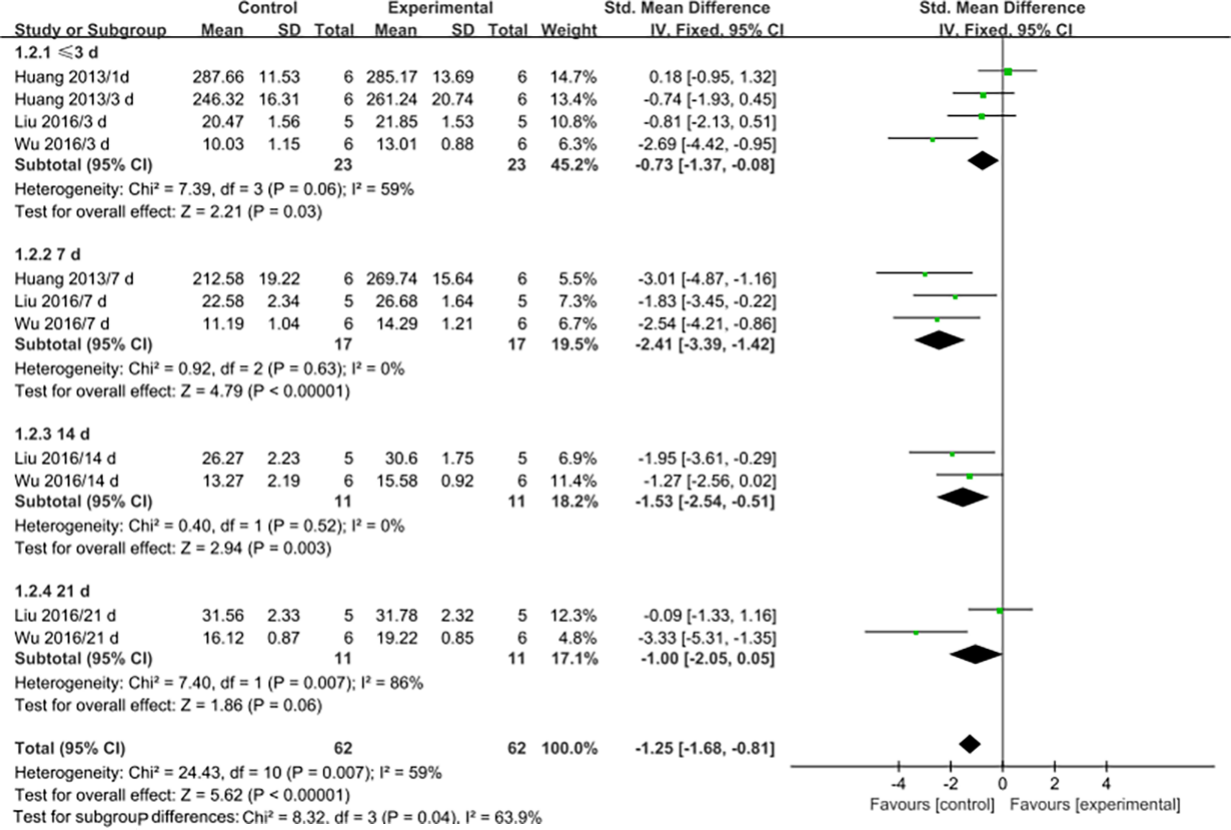
Forest plots showing the effects of MSCs on SOD levels. Control: PQ group; Experimental: MSC treatment group; CI: confidence interval; IV: independent variable; SD: standard deviation.

Serum or plasma SOD levels were assessed at 3 d after MSC transplantation or earlier in four studies [24,26,27], at 7 d after transplantation in three studies [24,26,27], at 14 d after transplantation in two studies [26,27], and at 21 d after transplantation in two studies [26,27] (Table 2). Generally, MSC therapy was associated with significantly increased SOD levels at 3 d or earlier (SMD: -0.73, 95% CI: -1.37 to -0.08, P = 0.03), 7 d (SMD: -2.41, 95% CI: -3.39 to -1.42, P<0.00001) and 14 d after transplantation (SMD: -1.53, 95% CI: -2.54 to 0.51, P = 0.003). However, no difference was observed at 21 d after transplantation (SMD: -1.00, 95% CI: -2.05 to 0.05, P = 0.06). Significant heterogeneity was observed at 3 d or earlier and 21 d after transplantation (I^2^ = 59% and 86%). The heterogeneity was mainly associated with the time point (Fig 3).

**Fig 4:**
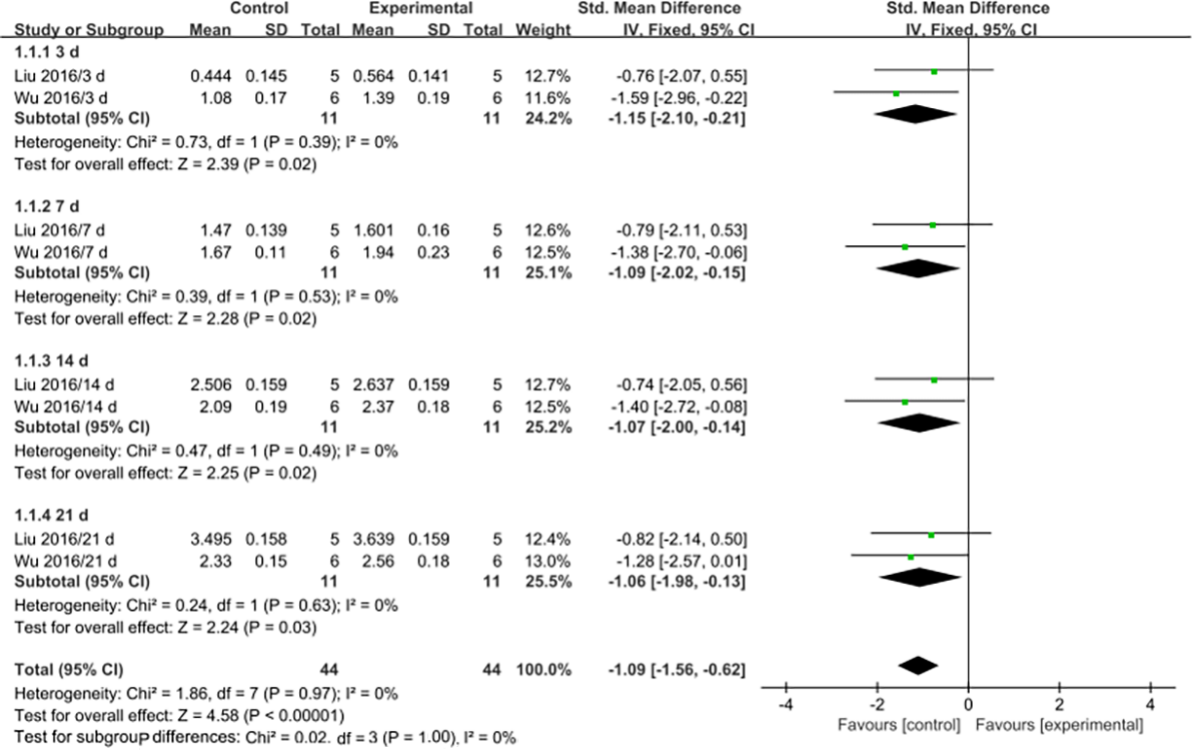
Forest plots showing the effects of MSCs on GSH levels. Control: PQ group; Experimental: MSC treatment group; CI: confidence interval; IV: independent variable; SD: standard deviation.

Serum or plasma GSH levels were assessed at 3 d after MSC transplantation or earlier in two studies [26,27], at 7 d after transplantation in two studies [26,27], at 14 d after transplantation in two studies [26,27], and at 21 d after transplantation in two studies [26,27] (Table 2). Generally, MSC therapy was associated with significantly increased GSH levels at 3 d (SMD: -1.15, 95% CI: -2.10 to -0.21, P = 0.02) 7 d (SMD: -1.09, 95% CI: -2.02 to -0.15, P = 0.02), 14 d (SMD: -1.07, 95% CI: -2.00 to -0.14, P = 0.02) and 21 d after transplantation (SMD: -1.06, 95% CI: - 1.98 to -0.13, P = 0.03). No significant heterogeneity was observed in the groups, and the findings were highly stable (Fig 4).

GSH-PX was measured in two studies (Table 2) [19,22]. The study by Xiong et al. [19] showed that plasma GSH-PX was decreased at 3 d after transplantation but increased at 7 d after transplantation, and no significant difference was observed at 14 d after transplantation. The study by Xiong et al. [19] showed that GSH-PX in lung tissue homogenates was increased at 72 h after transplantation.

**Fig 5:**
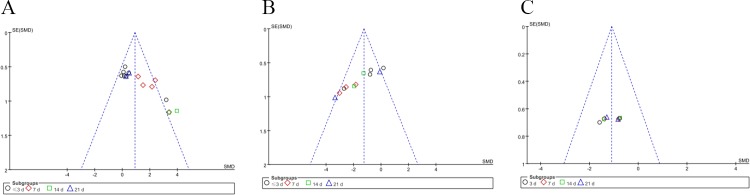
**Funnel Plot of MDA (A), SOD (B) and GSH (C) Data.** SE: standard error; SMD: standard mean difference.

A funnel plot of the MDA, SOD and GSH data showed that the values were distributed around the overall estimate, with no obvious publication bias (Fig 5). In addition, we present the MDA, SOD and GSH data in clear histogram format (S1 File).

### The effects of MSCs on the inflammatory response

Blood serum or plasma IL-1β was measured in four studies (Table 2). IL-1β levels were significantly decreased by MSC therapy at 7 d after transplantation [19,24,27]. Some studies showed no significant effect on IL-1β expression at 12 h [28], 24 h [28], 3 d [19], and 14 d [19,24] after transplantation, while others showed that IL-1β decreased at 48 h [28], 1 day [24], 3 d [24], 14 d [27], and 21 d [27] after transplantation.

Blood serum or plasma TNF-α was measured in six studies (Table 2). TNF-α levels were significantly decreased by MSC therapy at 7 d after transplantation [19,23,24,26,27]. Some studies showed no significant effect on TNF-α at 12 h [28], 24 h [28], 1 day [23], 3 d [19,26], and 14 d [19,24] after transplantation, while others showed that TNF-α decreased at 48 h [28], 1 day [24], 3 d [23,24,27], 14 d [26,27], and 21 d [26,27] after transplantation.

Blood serum or plasma TGF-β1 levels were measured in two studies (Table 2). No significant effect on TGF-β1 was observed at 21 d after transplantation [26], but its expression decreased at 3 d [26], 7 d [26], 14 d [26], and 28 d after transplantation [29]. Lung TGF-β1 expression decreased at 7 d, 14 d, and 28 d after transplantation [25].

Lung hydroxyproline was assessed in two studies (Table 2) [25,29]. Lung hydroxyproline levels were decreased by MSCs at 14 and 28 d after transplantation, but no significant difference was observed at 7 d after transplantation.

## Discussion

Hemodialysis, or blood filtration, to remove toxins is the primary method of treating PQ poisoning in the clinic [30]. In addition, drugs such as adiponectin [31], sodium ferulate combined with oxymatrine [32] and pirfenidone plus prednisolone [33] are being studied to treat lung injury, but the therapeutic effects are not ideal. This study summarized preclinical data describing the use of MSCs for the treatment of PQ poisoning in animal models of lung injury. MSCs derived from bone marrow, fat and umbilical cord were used to treat PQ poisoning in animal models within 24 h of PQ administration. The oxidative stress response, inflammatory response, survival status and histopathology of experimental animals were improved by MSCs.

The shortcomings of experimental reports in the included studies were determined by using the SYRCLE Risk of Bias tool. None of the 11 studies included was assessed as having a low risk of bias based on all of the reporting data entries included in the tool. Among all studies, a low risk of bias was assigned only if detailed, specific methodological descriptions were included. In other words, the appropriate methodology may be applied in these studies, but there are no explicit methodological descriptions in the articles. Our review suggests that it is imperative to improve description of the experimental methods, especially in preclinical studies of MSC therapy for PQ poisoning. The SYRCLE Risk of Bias tool may be a good reference tool to allow preclinical studies to reduce bias. In addition, according to the summary of MSC study characteristics (S3 Table), four of the studies did not report the markers and differentiation capability of the MSCs used. We also suggest that the criteria and general methodology for MSCs described in future studies must be more elaborate; greater article stringency will help to optimize conversion studies.

Large amounts of oxygen free radicals and MDA are produced during PQ-induced lung damage, resulting in the oxidation of membrane lipids, damage to pulmonary vascular endothelial cells, and the destruction of cellular barriers. SOD and GSH are important free radical scavengers. Quantitative analysis showed that MSCs significantly reduce MDA content in the serum or plasma of PQ-poisoned animals at 7 and 14 d after transplantation, but no significant differences are observed 3 d after transplantation or earlier or 21 d after transplantation. By contrast, serum or plasma SOD and GSH levels increase significantly after MSC transplantation at 3 d or earlier, 7 d or 14 d. This finding suggests that MSC administration reduces MDA levels by promoting serum or plasma SOD and GSH levels within 14 d, reducing oxidative stress and protecting lung tissues. In addition, blood MDA levels in the MSC treatment groups do not significantly different from those in the PQ group at or before 3 d after MSC transplantation, potentially because the transplanted MSCs have not yet arrived at their “niche” location to exert anti-oxidative effects. Moreover, PQ poisoning results in the development of fibrotic lesions in the target organ by 21 d after poisoning, and MDA levels primarily reflect oxidative stress in the acute phase; this may explain why there is no obvious difference in MDA content between the PQ and MSC treatment groups at 21 d.

Pro-inflammatory and pro-fibrogenic cytokines are closely associated with the initial and developmental stages of PQ-induced pulmonary fibrosis [34]. PQ activates the NF-κB signaling pathway, resulting in the release of TNF-α, IL-1β, and IL-6 and leading to acute lung injury [35]. IL-1β inhibits fluid transport across the distal lung epithelium, resulting in surfactant abnormalities and increased protein permeability across the alveolar-capillary barrier, playing a key role in the development of acute PQ-induced lung injury [36]. TNF-α is produced by alveolar macrophages during the early stage of lung injury and the later fibrosis stage, triggering the release of various pro-inflammatory cytokines and leading to fibroblast proliferation [37]. In addition, TNF-α causes cell death and alveolar epithelial dysfunction [38]. TGF-β promotes collagen deposition and the differentiation of fibroblasts into myofibroblasts and inhibits fibroblast autophagy [39,40]. Our review found that IL-1β, TNF-α and TGF-β1 levels in blood or lung tissues are decreased to differing degrees by MSCs within 28 d, suggesting that transplanted MSCs modulate inflammatory cytokines in the blood or local lung tissues via paracrine secretion functions and thus play anti-inflammatory and anti-fibrosis roles in the lung. In addition, hydroxyproline content in lung tissues is decreased by MSCs at 14 d and 28 d after transplantation, indicating that MSC administration decreases the collagen content in lung tissue. This result also confirms that MSCs exert an anti-fibrosis effect.

Interestingly, a single clinical study named “Human umbilical cord derived mesenchymal stem cell therapy in PQ poisoning induced lung injury” is shown on clinicaltrials.gov. The status of this study is unknown, and the notes for this study state: “Study has passed its completion date and status has not been verified in more than two years”. According to the clinical situation during PQ poisoning, the status quo of sponsors and collaborators, and the progression of Chinese stem cell research, we infer that there are three main reasons for the study status given above. First, PQ poisoning demonstrates acute progression in clinical practice, with respiratory failure usually occurring within 2 or 3 weeks, and large doses of PQ (> 40 mg/kg) cause multiple organ failure within days. Meanwhile, MSC preparation also requires adequate time. Patients usually receive emergency treatment to block the absorption of toxic substances in the short term. Therefore, there are certain difficulties associated with patient recruitment for this study. Second, the sponsors and collaborators shown in the study details are “Affiliated Hospital to Academy of Military Medical Sciences” and “Ivy institute of stem cells Co. Ltd”. After searching on the Internet (https://xin.baidu.com/detail/compinfo?pid=llHczwJVAmS4YZhuGUSXktjSo77kpo*crgkh&from=ps&tab=changeRecord), we found that the business scope and legal representation of this company have changed since May 2015, the initial start date of the study. Therefore, changes to the company's operating policies may constitute a commercial factor affecting enrollment. Third, the implementation of stem cell translational research policy is very important to standardize clinical research involving stem cells in China. An official document designated “Notification of the management of stem cell clinical research (trial)” was published in August 2015, and relevant policies are currently being updated; all stem cell clinical research must follow these scientific standards. Therefore, patient recruitment, commercial sponsorship, and policy support should be carefully considered when performing clinical cell therapy transformation research for PQ poisoning.

This study has some limitations. First, there is a high incidence of PQ poisoning in China, and most of the preclinical studies in this review come from the Chinese literature. Meanwhile, it is necessary to improve detailed descriptions of experimental methods to reduce the risk of bias. Second, we quantitatively meta-analyzed the oxidative stress indices MDA, SOD and GSH and qualitatively analyzed factors such as inflammatory cytokines and fibrosis, but these factors do not completely simulate the response to PQ poisoning in humans. Third, sub-groups in the SOD quantitative analysis demonstrated an I^2^>50%, which may affect the credibility of the results. In addition, the MDA funnel chart shows that four studies fall outside of the 95% confidence interval and that the overall I^2^ value of MDA is 63%, suggesting that the moderate heterogeneity may be caused by these four studies, although the heterogeneity of each subgroup was acceptable. Fourth, the maximum time point we statistically analyzed in this study was 28 d after PQ poisoning. Whether MSCs directly differentiate into alveolar epithelial cells to prevent PQ-induced fibrosis and exert long-term therapeutic effects on lung regeneration is worthy of further exploration.

## Conclusion

Preclinical data regarding MSC treatment of PQ-induced lung injury showed that MSCs exerted therapeutic effects on animal models of lung injury induced by PQ poisoning; these effects may be associated with the reduction of oxidative stress and inflammatory cytokines. MSCs may be a new and effective biological agent for the treatment of clinical PQ poisoning, and our review serves to augment the rationale for clinical studies.

## Supporting information

S1 Table PRISMA checklist. (DOC)

S2 Table SYRCLE risk of bias assessment for included studies. (DOCX)

S3 Table MSC criteria and general methodology in the included studies. (DOCX)

S4 Table Literature search terms (Used in PubMed). (DOCX)

S1 File Histogram of MDA, SOD and GSH in the included studies. (DOCX)
