# Supplementary material for: Mesenchymal stem cell therapy for paraquat poisoning: A systematic review and meta-analysis of preclinical studies
Source: PLoS One. 2018 Mar 22;13(3):e0194748. doi: 10.1371/journal.pone.0194748 (PMC5864035; doi:10.1371/journal.pone.0194748)
Supplement: S2 Table — (DOCX) [file pone.0194748.s002.docx]

**S2 Table.** **SYRCLE Risk of Bias Assessment for the Included Studies**.

| Author (Year) | Random sequence generation? | Groups similar at baseline? | Allocation concealed? | Animals randomly housed? | Blinding of caregivers and/or examiners? | Random selection for outcome assessment? | Blinding of outcome assessor? | Incomplete outcome data addressed? | Free from selective outcome reporting? | Free from other bias? |
| --- | --- | --- | --- | --- | --- | --- | --- | --- | --- | --- |
| Xiong et al. (2014) [19] | Unclear | Yes | Unclear | Unclear | Unclear | Unclear | Yes | Yes | Yes | Yes |
| Gao et al. (2011) [22] | Unclear | Yes | Unclear | Unclear | Unclear | Unclear | Yes | Yes | Yes | Yes |
| Chen et al. (2016) [25] | Yes | Yes | Unclear | Unclear | Unclear | Unclear | Yes | Yes | Yes | Yes |
| Huang et al. (2012) [23] | Unclear | Yes | Unclear | Unclear | Unclear | Unclear | Yes | Yes | Yes | Yes |
| Huang et al. (2013) [24] | Unclear | Yes | Unclear | Unclear | Unclear | Unclear | Yes | Yes | Yes | Yes |
| Zhang et al. (2011) [29] | Unclear | Yes | Unclear | Unclear | Unclear | Unclear | Yes | Yes | Yes | Yes |
| Wu et al. (2016) [27] | Yes | Yes | Unclear | Unclear | Unclear | Unclear | Yes | Yes | Yes | Yes |
| Lü et al. (2014) [20] | Unclear | Yes | Unclear | Unclear | Unclear | Unclear | Unclear | Yes | Yes | Yes |
| Liu et al. (2016) [26] | Unclear | Yes | Unclear | Unclear | Unclear | Unclear | Yes | Yes | Yes | Yes |
| Wu et al. (2017) [28] | Unclear | Yes | Unclear | Unclear | Unclear | Unclear | No | Yes | Yes | Yes |
| Tsai et al. (2013) [21] | Unclear | Yes | Unclear | Unclear | Unclear | Unclear | Yes | Yes | Yes | Yes |
