# Supplementary material for: Mesenchymal stem cell therapy for paraquat poisoning: A systematic review and meta-analysis of preclinical studies
Source: PLoS One. 2018 Mar 22;13(3):e0194748. doi: 10.1371/journal.pone.0194748 (PMC5864035; doi:10.1371/journal.pone.0194748)
Supplement: S3 Table — (DOCX) [file pone.0194748.s003.docx]

**S3 Table. MSC Criteria and General Methodology in the Included Studies.**

| Author (year) | Were MSCs purchased or supplied? | Plastic adherence? | Positive markers | Negative markers | Differentiation capability | Cell expansion media | Passage number |
| --- | --- | --- | --- | --- | --- | --- | --- |
| Xiong et al. (2014) [19] | No | Yes | CD44, CD166 | CD34, CD45 | Not reported | DMEM/F12, 10% FBS | 3 |
| Gao et al. (2011) [22] | No | Yes | CD71, CD44 | CD34, CD45 | Osteocytes | DMEM | 3 |
| Chen et al. (2016) [25] | Not reported | Not reported | Not reported | Not reported | Not reported | Not reported | Not reported |
| Huang et al. (2012) [23] | No | Yes | CD44, CD166 | CD34, CD45 | Not reported | DMEM/F12, 10% FBS | 3 |
| Huang et al. (2013) [24] | No | Yes | CD44, CD166 | CD34, CD45 | Not reported | DMEM/F12, 10% FBS | 3 |
| Zhang et al. (2011) [29] | No | Yes | CD90 | CD34, CD45 | Adipocytes, osteocytes | LG-DMEM, 20% FBS, penicillin, streptomycin | 4 |
| Wu et al. (2016) [27] | Not reported | Yes | Not reported | Not reported | Not reported | DMEM | Not reported |
| Lü et al. (2014) [20] | No | Yes | Not reported | Not reported | Not reported | DMEM | 6 to 8 |
| Liu et al. (2016) [26] | Not reported | Yes | Not reported | Not reported | Not reported | DMEM, 10% FBS | Not reported |
| Wu et al. (2017) [28] | No | Yes | CD29, CD90, CD105 | CD31, CD34 | Adipocytes, osteocytes | DMEM/F12, 10% FBS | 3 |
| Tsai et al. (2013) [21] | No | Yes | CD29, CD44, CD71, CD73, CD90, CD105, SH-2, SH-3 | CD13, CD34, CD45, CD133 | Adipocytes, osteocytes, chondrocytes, hepatocytes | IMDM, 10% FBS, EGF, bFGF, penicillin, streptomycin, L-glutamine | 5 to 13 |
| **Abbreviations**: Dulbecco’s modified Eagle’s medium (DMEM); fetal bovine serum (FBS); Iscove's modified Dulbecco's medium (IMEM); epidermal growth factor (EGF). | | | | | | | |
