# Supplementary material for: Mesenchymal stem cell therapy for paraquat poisoning: A systematic review and meta-analysis of preclinical studies
Source: PLoS One. 2018 Mar 22;13(3):e0194748. doi: 10.1371/journal.pone.0194748 (PMC5864035; doi:10.1371/journal.pone.0194748)
Supplement: S4 Table — (DOCX) [file pone.0194748.s004.docx]

**S4 Table. Literature Search Terms (Used in PubMed)**.

(((((((((((((((((((((((((((((((((((((Cell, Mesenchymal Stromal[Title/Abstract]) OR Cells, Mesenchymal Stromal[Title/Abstract]) OR Mesenchymal Stromal Cell[Title/Abstract]) OR Stromal Cell, Mesenchymal[Title/Abstract]) OR Stromal Cells, Mesenchymal[Title/Abstract]) OR Mesenchymal Stem Cells[Title/Abstract]) OR Cell, Mesenchymal Stem[Title/Abstract]) OR Cells, Mesenchymal Stem[Title/Abstract]) OR Stem Cell, Mesenchymal[Title/Abstract]) OR Mesenchymal Progenitor Cells[Title/Abstract]) OR Mesenchymal Stromal Cells, Multipotent[Title/Abstract]) OR Stem Cells, Mesenchymal[Title/Abstract]) OR Multipotent Mesenchymal Stromal Cells[Title/Abstract]) OR Mesenchymal Stem Cell[Title/Abstract]) OR Mesenchymal Progenitor Cell[Title/Abstract]) OR Cell, Mesenchymal Progenitor[Title/Abstract]) OR Cells, Mesenchymal Progenitor[Title/Abstract]) OR Progenitor Cell, Mesenchymal[Title/Abstract]) OR Progenitor Cells, Mesenchymal[Title/Abstract]) OR Bone Marrow Stromal Cells, Multipotent[Title/Abstract]) OR Multipotent Bone Marrow Stromal Cells[Title/Abstract]) OR Bone Marrow Stromal Cells[Title/Abstract]) OR Bone Marrow Stromal Cell[Title/Abstract]) OR Wharton Jelly Cells[Title/Abstract]) OR Cells, Wharton Jelly[Title/Abstract]) OR Wharton's Jelly Cells[Title/Abstract]) OR Cell, Wharton's Jelly[Title/Abstract]) OR Cells, Wharton's Jelly[Title/Abstract]) OR Jelly Cell, Wharton's[Title/Abstract]) OR Wharton's Jelly Cell[Title/Abstract]) OR Whartons Jelly Cells[Title/Abstract])) OR "Mesenchymal Stromal Cells"[Mesh])) AND Pulmonary) AND Paraquat)) NOT ((review[Publication Type]) OR review literature as topic[MeSH Terms])
