# Supplementary figures and images for: Mesenchymal stem cell therapy for paraquat poisoning: A systematic review and meta-analysis of preclinical studies
Source: PLoS One. 2018 Mar 22;13(3):e0194748. doi: 10.1371/journal.pone.0194748 (PMC5864035; doi:10.1371/journal.pone.0194748)

**S1 File. Histogram of MDA, SOD and GSH in the Included Studies.**


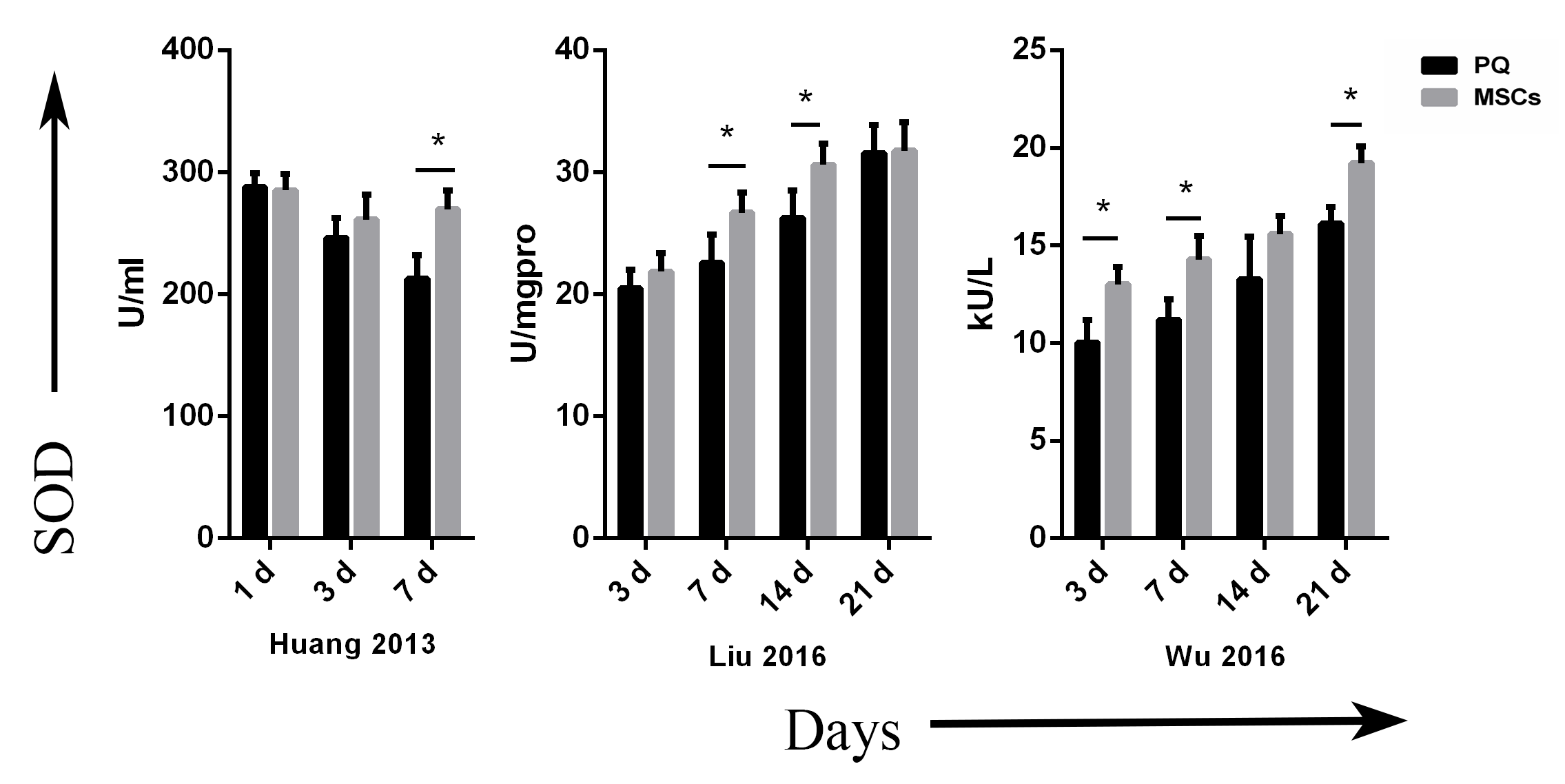

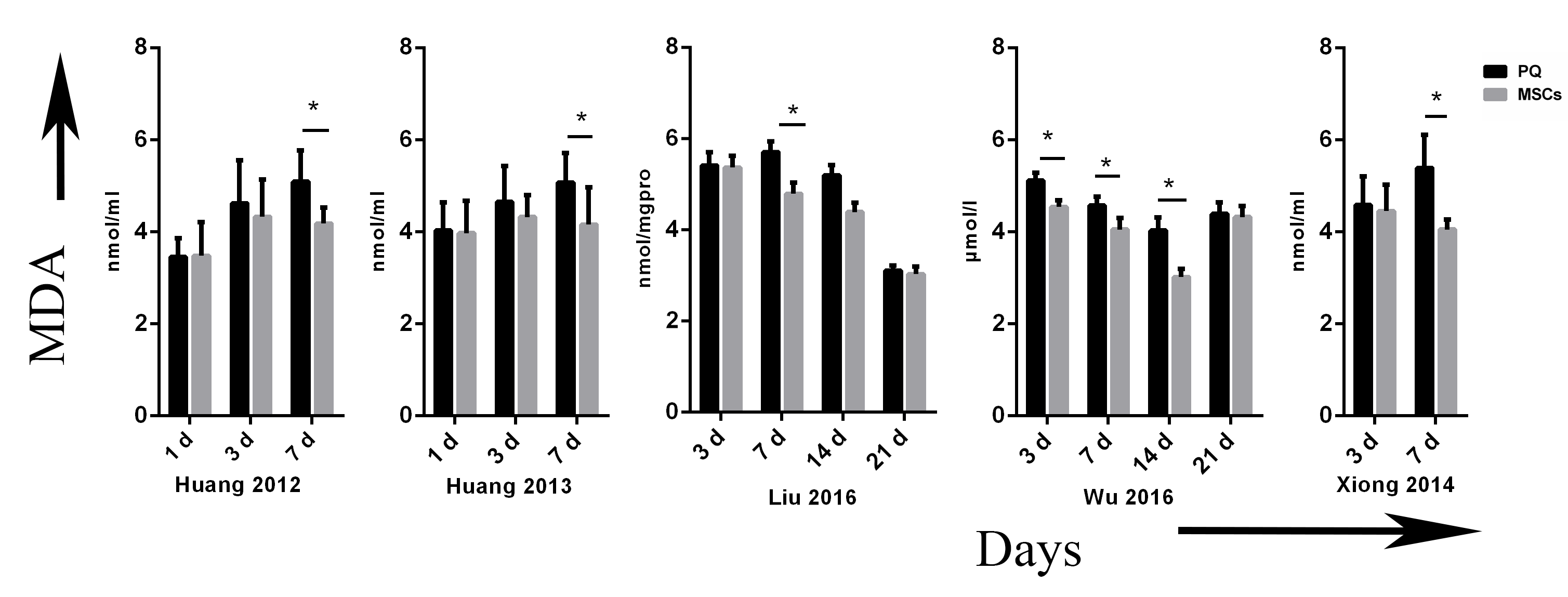

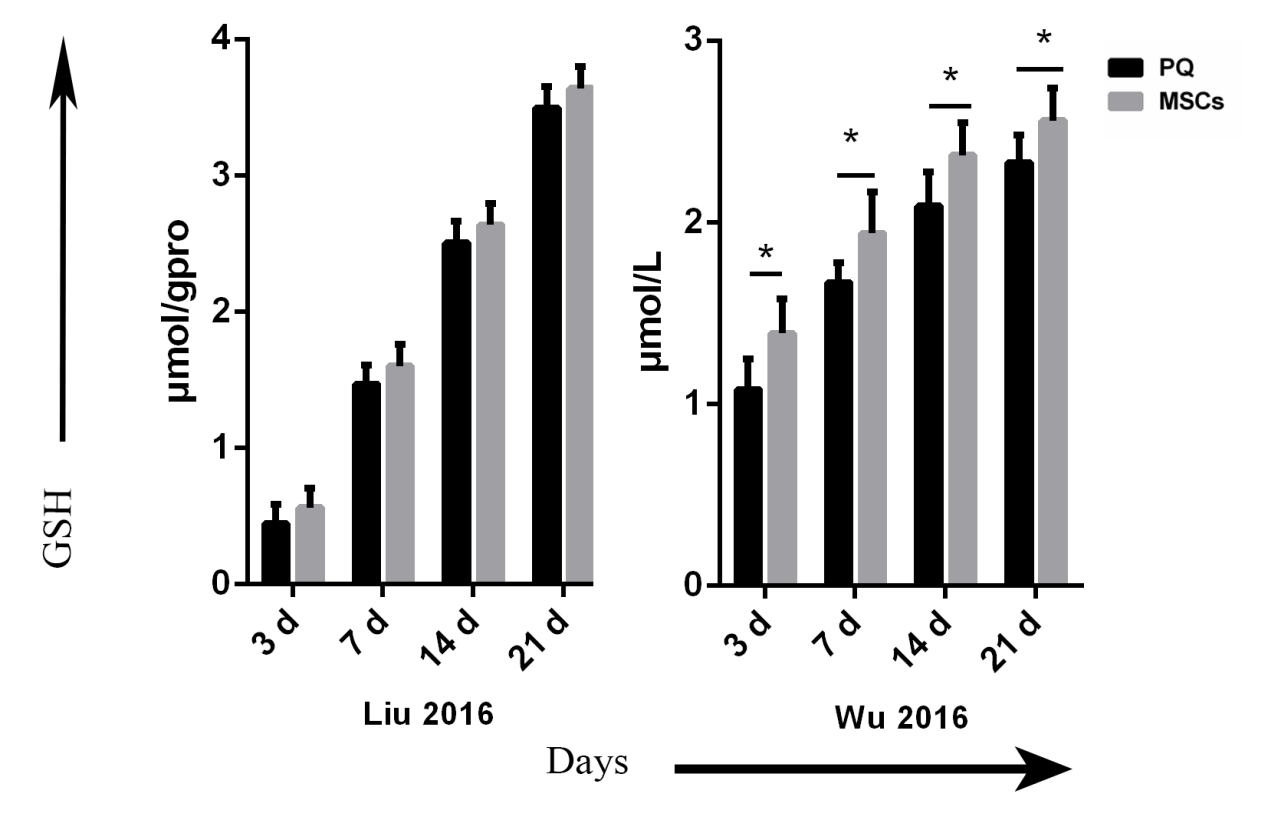

Supplement: S1 File — (DOCX) [file pone.0194748.s005.docx]
